# Resveratrol protects against ICV collagenase-induced neurobehavioral and biochemical deficits

**DOI:** 10.1186/s12950-017-0158-3

**Published:** 2017-06-09

**Authors:** Navdeep Singh, Yashika Bansal, Ranjana Bhandari, Lovish Marwaha, Raghunath Singh, Kanwaljit Chopra, Anurag Kuhad

**Affiliations:** Pharmacology Research Laboratory, University Institute of Pharmaceutical Sciences, UGC-Centre of Advanced Study, Panjab University, Chandigarh, 160 014 India

**Keywords:** Intracerebral hemorrhage, Stroke, Resveratrol, Collagenase

## Abstract

**Background:**

Indeed, intracerebral hemorrhage (ICH) account for only 15% of all strokes but it is one of the most devastating subtype of stroke associated with behavioral, cognitive and neurological deficits. The primary cause of neurological deficits in ICH is the hematoma growth, generation of free radicals, inflammatory cytokines and exhausting endogenous anti-oxidant machinery. It has been found that neuroinflammation following ICH leads to exaggeration of hallmarks of ICH. With this background, the study was aimed to evaluate the protective effect of resveratrol (RSV) in intracerebroventricular (ICV) collagenase (COL) induced neurological deficits in rats.

**Methods:**

The present study was designed to explore the protective effects of resveratrol (5, 10, 20 mg/kg) against ICV-COL induced ICH. Animals were subjected to a battery of behavioral tests to access behavioral changes, including neurological scoring tests (cylinder test, spontaneous motility, righting reflex, horizontal bar test, forelimb flexion), actophotometer, rotarod, Randall Sellito and von Frey. Post stroke depression was estimated using forced swim test (FST). Memory deficit was monitored using Morris water maze (MWM).

**Results:**

Chronic treatment with RSV (20 mg/kg) for 21 days restored various behavioral changes, including neurological scoring tests (cylinder test, spontaneous motility, righting reflex, horizontal bar test, forelimb flexion), actophotometer, rotarod, Randall Sellito and Von Frey. RSV also restores increase in immobility time forced swim test used to evaluate post stroke depression and impaired memory deficit in Morris water maze. RSV administration also attenuated increased nitro-oxidative stress and TNF-α level. RSV being a potent antioxidant also restores changes in endogenous anti-oxidant levels.

**Conclusion:**

In conclusion, our research demonstrates that RSV has a protective effect against ICH by virtue of its anti-inflammatory property and antioxidant and nitrosative stress restoring property.

## Background

Intracerebral hemorrhage (ICH) is an intracranial rupture of small blood vessels in the brain parenchyma with persistent increased blood pressure. Hypertension is the most common cause of ICH [1]. Primary ICH, with no underlying vasculopathy, is associated with about 10–15% of all stroke cases, whereas in Oriental population, the figures are as up to 30–50% [2–5]. Despite of the previous understanding of the mechanisms associated with ICH, there is no FDA approved treatment for ICH till date. ICH injury occurs in two different steps. First the leakage of blood in brain parenchyma elevates intracranial pressure that causes mechanical shear and damage to the neurons and glial cells [6]. Secondly, products of erythrocyte lysis and damaged microglia trigger the inflammatory cascade and oxidative stress, causing secondary damage associated with ICH [7]. Oxidative stress and neuronal cell damage associated cognitive decline are the most important implications in the pathogenesis of ICH [8–10]. In the delayed phase of ICH, noxious proteases like matrix metalloproteinase (MMP-9) which are released pathologically from neutrophils and activated microglia [11] cause structural damage to the blood brain barrier [12] leading to the brain edema. Release of erythrocyte lysis product like iron, which catalyzes the production of free radicals and reactive oxygen species (ROS), is the key mediator of inflammatory cascade leading to cell death and perihematomal edema [13, 14]. An increase in oxidative stress saturates like reduced glutathione (GSH), which is the main antioxidant molecule in brain, along with other endogenous antioxidants like superoxide dismutase (SOD), catalase etc. Although endogenous antioxidants can scavenge and neutralize the free radicals. Hence, neuroprotection in ICH needs a combined approach of decreasing different byproducts of secondary brain damage and restoring the antioxidant machinery in the brain. ICH leads to neuroinflammation and mitochondrial dysfunction, which are key features of chronic neurodegenerative diseases. These conditions leads to increased oxidative stress by excessive release of harmful reactive oxygen and nitrogen species (ROS and RNS), which further promote neuronal damage and subsequent inflammation resulting in a feed-forward loop of neurodegeneration. The cytokine tumor necrosis factor-α (TNF-α), a master regulator of the immune system, plays an important role in the propagation of inflammation due to the activation and recruitment of immune cells via its receptor [15].

Animal model of ICH have been used to study the pathophysiology and treatment of ICH, including the microballoon model, the bacterial COL injection model and the autologous blood injection model. In the COL injection model, the hemorrhage size is controllable which was induced by small vessel breakdown. This model also can mimic the onset of spontaneous intraparenchymal bleeding and the expansion of continuous bleeding in ICH patients. In the past several years, previous studies have proven that our modified COL IV injection model is a reliable and reproducible model of ICH in rat [16].

Antioxidants and anti-inflammatory drugs have been evaluated for potential pharmacotherapeutic activity in different models of ischemic and subarachnoid hemorrhage [17–19] but no effective treatment method has been resolved out. So, agents that can strengthen endogenous antioxidant machinery and have direct ROS scavenging properties are the pressing need of the hour against ICH associated oxidative damage. RSV, a natural polyphenol found in grapes and red wine, could be a phytochemical of choice for ICH related oxidative damage. It has been shown to possess potent anti-oxidative, anticancer, anti-inflammatory and antiapoptotic effects in animal and clinical studies [20–22]. Moreover, recent evidences indicate angiogenic and protective effects of RSV in variety of in-vivo and in-vitro ischemic model [23–25]. Furthermore, accumulating evidences indicates potent neuroprotective and cardioprotective properties of RSV [26–28]. With this background, the current study was designed to explore pharmacotherapeutic potential of RSV in experimental paradigm of ICH.

## Methods

### Animals

Adult female Wistar rats (200-230 g) bred in the Central Animal House facility of Panjab University, Chandigarh, India were used for the study. The animals had free access to standard rodent food pellets (Ashirwad Industries, Mohali, India) and water. They were acclimatized to the laboratory conditions before the experiment. All the experiments were conducted between 9 am to 5 pm. The experimental protocols were approved by the Institutional Animal Ethics Committee (IAEC) (PU/IAEC/S/14/94) of Panjab University and conducted according to Committee for the Purpose of Control and Supervision on Experiments on Animals (CPCSEA) guidelines for the use and care of experimental animals.

### COL-induced ICH

Female Wistar rats (200–230 g) were anaesthetized using thiopentone sodium (Neon Laboratories, India, 45 mg/kg, i.p.) and placed into stereotactic frame. The head was shaved to expose the skull and a burr hole was made to introduce a calibrated Hamilton syringe into the center of the striatum (stereotactic coordinates from bregma: 0.2 mm posterior, 3.0 mm lateral, 6.0 mm underneath the dural surface) [29]. A solution containing 0.25 IU of COL, diluted in saline to an infusion volume of 1 μL was infused over 5 min. Burr hole was filled with dental cement and fixative after completion of the infusion. Daily application of Neosporin powder was done to prevent any infection. Sham animals were administered same volume of saline. Animals were fed with oral glucose for 4 days following surgery and then replaced with normal water.

### Drugs and treatment schedule

**Fig. 1:**
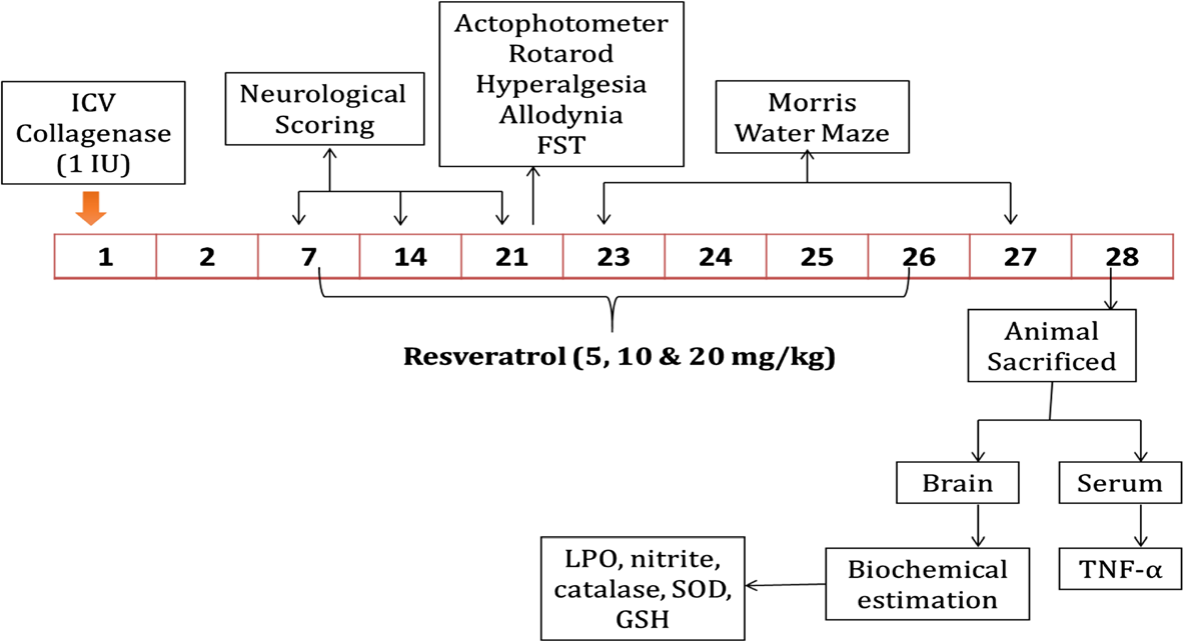
Diagrammatical representation of experimental design

COL and RSV were purchased from Sigma-Aldrich (St. Louis MO, USA). COL was dissolved in normal saline (0.9% NaCl) and administered ICV. RSV was dissolved in distilled water and administered by oral gavage from day 1 after COL administration till the end of the study i.e. 27 days (Fig. 1). Initially 40 animals were taken and surgery was done on 30 animals. The mortality rate was 12.5%. Animals were distributed randomly into 7 treatment groups consisting of 5 animals in each group. Group 1 & 2 were control and sham respectively given saline only. Group 3 was per se group treated with RSV (40 mg/kg, p.o.). Group 4 was ICH group treated with 0.25 IU COL-ICV. Group 4,5 & 6 were ICH-RSV group treated with three different doses i.e. 5, 10 and 20 mg/kg [30].

### Neurofunctional assessments

#### Spontaneous motility

The animal was tested for spontaneous motility and exploration of the surroundings, by putting it onto a clean empty surface in familiar surroundings. If animal failed to initiate motility after 10s, spontaneous motility score was given as 0. If animal moved within 10s, it was scored as 1 [31].

#### Horizontal bar test

Animal was suspended by its forelimbs on a bar having solid platforms on both ends. The bar was placed about 40 cm above the surface having a foamed surface to guarantee safe landing. The animal was expected to grasp the bar and to hang for 3 s [31].

**Method of scoring:**

If animal hangs with one forelimb, then score: 1.

If it hangs with both then Score: 2.

If it hangs with both forelimbs + one hindlimb: 3.

If it hangs with both forelimbs + both hindlimbs: 4.

If it falls before 3 s then Score: 0.

#### Righting reflex

The animal was held in supine position in the hand. The righting reflex is when animal return to its natural position and was scored 1. If the animal failed to return to normal position, it was scored 0 [31].

#### Forelimb flexion

The animal was suspended by its tail upside down toward the ground. Animal was expected to extend its forelimb toward the ground. A rat that had undergone hemorrhage flexed the contralateral forelimb and twisted its body towards the contralateral side of damage [31].

Score given was 1, if animal extended its forelimb towards ground and 0 if animal flexed its limb toward contralateral to the ictus.

#### Rotarod

The rotarod apparatus consists of a rod which rotates at a constant speed (25 rpm). Animals with hemorrhagic damage tend to fall earlier than animal with no hemorrhagic insult.

#### Method of scoring

Fall-off time for 5 min was recorded.

#### Cylinder test

The test was used to evaluate the forelimb and vibrissae proprioception deficits in rodent ICH model. The exploratory behavior in rat is seen as a neuronal basis of spatial and motor behavior. A transparent plexiglass was used to evaluate the forelimb deficits. Typically, an animal tries to explore the vertical walls of the cylindrical plexiglass by rearing upon their hind limbs and using its forelimbs and vibrissae. Exploratory behavior was assessed; number of independent wall placements for the right forelimb, left forelimb and both fore limbs simultaneously is recorded. Animals with unilateral damage will fail to show symmetrical vertical explorations. The test can detect even mild neurological impairments [31, 32].

Total time for recording was 5 min and scored accordingly.

**Method of scoring:**

If Right forelimb was placed on cylinder wall then Score: 1.

If Left forelimb was placed on cylinder wall then Score: 1.

If both forelimbs were placed on cylinder wall then Score: 2.

No rearing and exploration Score: 0.

#### Forced swim test

This test was used to evaluate post stroke depression in rats. The animals were allowed to swim in a jar (60×30×45 cm) filled with water upto a height of about 40 cm so that animal could not touch its tail to the bottom to take support. The animal would try to escape the aversive stimuli for first few minutes by vigorous swimming but became passive later on by showing very little mobility. An animal is considered to be immobile whenever it remained floating passively in the water in a slightly hunched but upright position, its nose above the water surface. The total immobility time was recorded for last 4 minin the total test of 6 min on 14th day after COL administration [33].

#### Assessment of mechanical hyperalgesia

Mechanical hyperalgesia was estimated using Randall-Sellito (IITC Life Science, Woodland Hills, CA). Pressure was increased gradually from 10 g/s, with the cut-off of 250 g to avoid any injury. Measurements were taken from the contralateral hindpaw and readings were expressed in mass units (gram). Three tests separated by at least 15 min were performed for each animal on day 21 after COL administration in caudate nucleus and the mean value of these tests was calculated [34].

#### Assessment of allodynia

Quantitative assessment of allodynia was done in hindpaws by stimulation with flexible von Frey filaments and the withdrawl threshold was estimated. Rats were placed in individual plexiglass boxes on a stainless-steel mesh floor and were allowed to acclimatize for at least 30 min. A series of calibrated von Frey filament (IITC Life Science, Woodland Hills, CA) was applied perpendicularly to the plantar surface of the hind paw with sufficient force. Brisk withdrawal or paw flinching was considered as a positive response. The test was repeated three to four times and mean value was reported [34].

#### Morris water maze test

Morris water maze (MWM) test is a behavioral and spatial learning test mostly used for rodents and involves water navigation task. It is mostly used to assess the spatial memory [35, 36]. The MWM consist of a large circular pool (150 cm in diameter, 45 cm in height, filled to a depth of 30 cm with water) maintained at temperature 28 ± 1 °C where the rodent is supposed to find an invisible or visible platform. The circular pool was divided into four equal quadrants and a submerged platform was placed 1 cm below the level of water unaltered in the middle of the target quadrant. During the test trial the individual rat was gently put into the water in one of the four starting positions of the divided quadrant (which is to be selected randomly) and was allowed to locate the submerged platform. Once the animal locates the platform it was allowed to stay there for the next 20 s. The cut-off time for the animal to locate the platform is 90 s. If the animal fails to achieve the platform it was gently guided onto the platform and was allowed to stay there for 20 s. The MWM task was carried out for five consecutive days (23-27th day) where the animals were subjected to training trials, each at an interval of 30 min approximately for days and probe test was done on 27th day. The latency time to escape and locate the platform in water maze was noted as an index of acquisition or learning using a computer tracking system with Ethiovision software (Noldus Information Technology, Wageningen, Netherlands).

#### Maze retention probe trial

To assess the extent of memory consolidation a probe trial was performed wherein the animal was placed into the pool for a total duration of 90 s as in the training trial, without the availability of the hidden platform. The probe trial was performed 24 h after the last training period (on day 27st). Parameters like time spent in the target quadrant and frequency of appearance in the target quadrant was calculated using computer tracking system with Ethiovision software (Noldus Information Technology, Wageningen, The Netherlands) which indicated the degree of memory consolidation that has taken place after learning [37].

#### Assessement of total locomotor activity

To assess the effect of locomotor activity, animals were subjected to a period of 5 mins test using digital actophotometer [IMCORP, Ambala] which consist of a square (30 × 30 cm) closed arena equipped with 12 infrared light sensitive photocells in two rows (six in each row), at a distance of 3 and 9 cm respectively. Animals were placed individually in the activity chamber for a 3-min habituation, and after that the readings were calculated for another 5 mins. Locomotor activity was expressed in terms of total photo beam counts for 5 mins per mouse. It was ensured that the room is sound and light attenuated to get accurate readings [38].

### Biochemical estimation

#### Brain homogenate preparation

The whole brain samples were rinsed with ice cold saline (0.9% sodium chloride) and homogenized in chilled phosphate buffer (pH 7.4). The homogenate was centrifuged at 800 g for 5 min at 4^0^ C to separate the nuclear debris. The supernatant thus obtained was centrifuged at 10,500 g for 20 min at 4 °C to get the post mitochondrial supernatant, which was used to assay lipid peroxidation, nitrite, reduced glutathione, superoxide dismutase and catalase activity.

#### Lipid peroxidation

The extent of lipid peroxidation was determined quantitatively in the form of thiobarbituric acid-reactive substances by the method described by the [39]. Briefly, 0.5 ml of Tris–HCl was added to 0.5 ml of post-mitochondrial supernatant and was incubated at 37 °C for 2 h. After incubation, 1 ml of 10% trichloroacetic acid was added and centrifuged at 300gfor 10 min. Then, 1 ml of 0.67% thiobarbituric acid was added to the tubes containing 1 ml of supernatant and the tubes were kept in boiling waterfor 10 min. After cooling, 1 ml of double distilled water wasadded, and absorbance was measured at 532 nm (PERKIN ELMER UV/VIS Spectrophotometer, Lamda 20). The amount of malondialdehyde was calculated using molarextinction coefficient of 1.56 × 10^5^ M^−1^ cm-^1^ and expressedas nanomole of malondialdehyde equivalents per milligramprotein.

#### Reduced glutathione

Reduced glutathione was assayed by the method described by Jollow DJ, Mitchell JR, Zampaglione N and Gillette JR [40]. 1.0 ml of postmitochondrial supernatant (10%) was precipitated with 1.0 ml of sulfosalicylic acid (4%). The samples were kept at 4 °C for at least 1 h and then centrifuged at 1200 rpm for 15 min at same temperature. The assay mixture contained 0.1 ml supernatant, 2.7 ml phosphate buffer (0.1 M, pH 7.4), and 0.2 ml 5,5′-dithiobis-(2-nitrobenzoic acid) (Ellman’s reagent, 0.1 mM, pH 8.0) in a total volume of 3.0 ml. The yellow colour developed was read at 412 nm (PERKIN ELMER UV/VIS Spectrophotometer, Lamda 20) and reduced glutathione levels were calculated using molar extinction coefficient of 1.36 · 10^4^ M^−1^ cm^−1^ and expressed as micromole per milligram protein.

#### Catalase activity

Catalase activity was determined by the method of [41]. Briefly, the assay mixture consisted of 1.95 ml phosphate buffer (0.05 M, pH 7.0), 1.0 ml hydrogen peroxide (0.019 M), and 0.05 ml postmitochondrial supernatant (10%) in a final volume of 3.0 ml. Changes in absorbance were recorded at 240 nm (PERKIN ELMER UV/VIS Spectrophotometer, Lamda 20) for 1 min. Catalase activity was quantified using the millimolar extinction coefficient of H_2_O_2_ (0.07 mM) and expressed as micromoles of H_2_O_2_ decomposed per minute per milligram protein.

#### Superoxide dismutase (SOD)

SOD activity was assayed by the method described by [42]. The assay system consists of EDTA 0.1 mM, sodium carbonate 50 mM and 96 mM of nitro blue tetrazolium (NBT). In the cuvette, 2 ml of the above mixture, 0.05 ml of hydroxylamine and 0.05 ml of the supernatant were added and auto-oxidation of hydroxylamine was measured for 2 min at 30 s intervals by measuring absorbance at 560 nm (PERKIN ELMER UV/VIS Spectrophotometer, Lamda 20).

#### Protein estimation

Protein concentration of each sample was determined using biuret method as described [43] by using bovine serum albumin as standard.

### Collection of blood samples

After the completion of behavioural parameters (32th day), blood samples were taken from retro-orbital plexus and animals were sacrificed by cervical dislocation. Serum was separated from the clotted blood kept at room temperature in the test tubes. Tubes were centrifuged at 3000 rpm for 10 min to separate serum later stored at −20 °C.

### Molecular estimation

#### TNF-α estimation

The quantification of TNF-α was done by the help and instructions provided by R&D Systems Quantikine rat TNF-α immunoassay kit. The intensity of the color was measured corresponds to the amount of rat TNF-α bound in the initial step. The sample values were then read off from the standard curve. Values were expressed as mean ± SEM.

#### Statistical Analysis

Statistical analysis was done using Graphpad prism software. One way ANOVA was used followed by Tukey’s multiple comparison tests. The values were expressed as mean ± SEM and *p* < 0.05 were considered statistically significant.

## Results

### Neurological scoring

#### Effect of RSV on neurological scorings in cylinder test

The cylinder test was used to evaluate rodent’s spontaneous forelimb use. There was significant reduction in scoring in cylinder test from 7th day onwards in COL treated rats as compared to the control rats, with maximum reduction at 14th day [F_(6,28)_ = 9.65 (*p* < 0.05)]. On 14th day RSV (10 mg/kg) showed improvement but was not significant but on 21st day, RSV (10 and 20 mg/kg) showed significant improvement in scoring as compared to COL treated group. However, RSV (5 mg/kg) did not produce any effect (Table 1).

**Table 1:** Effect of resveratrol (5, 10 and 20 mg/kg) on different neurological scoring tests on 21st day

Behavioral test	Horizontal Bar test	Cylinder Test	Spontaneous motility	Forelimb flexion	Righting reflex
Group					
Control	4.0 ± 0.0	43.4 ± 6.9	1.0 ± 0.0	1.0 ± 0.0	1.0 ± 0.0
Sham	3.8 ± 0.4	42.8 ± 2.9	0.8 ± 0.2	1.0 ± 0.0	1.0 ± 0.0
Control +RSV (20 mg/kg)	3.8 ± 0.4	41.6 ± 2.8	1.0 ± 0.0	1.0 ± 0.0	1.0 ± 0.0
ICV-collagenase (1 IU)	0.6 ± 0.4^#^	10.3 ± 3.0^#^	0.2 ± 0.2^#^	0.2 ± 0.2^#^	0.2 ± 0.0^#^
ICH+ RSV (5 mg/kg)	1.8 ± 0.4	23.8 ± 5.9	0.4 ± 0.2*	0.6 ± 0.2	0.0 ± 0.0
ICH + RSV (10 mg/kg)	2.8 ± 0.4*	34.2 ± 5.2*	0.8 ± 0.2*	0.6 ± 0.2	0.2 ± 0.2
ICH + RSV(20 mg/kg)	3.4 ± 0.4*	38.4 ± 6.2*	1.0 ± 0.0*	1.0 ± 0.0*	1.0 ± .00*^,$^

Values are expressed as mean ± SEM. ICH indicates ICV-collagenase treated rats; RSV, resveratrol. One way ANOVA followed by Tukey’s multiple comparison tests was applied. #*p* < 0.05 as compared to naïve group. *p < 0.05 as compared to ICV-collagenase group. $*p* < 0.05 as compared to ICH + RSV (5 mg/kg) group

#### Effect of RSV on scorings in horizontal bar test

Progressive reduction in neurological scoring was seen from day 7th to 21st with maximum reduction on 21st day [F_(6,28)_ = 1.856 (*p* < 0.05)]. Oral administration of RSV (10 & 20 mg/kg) significantly improved scoring as compared to the COL treated group although scoring remain unchanged in RSV (5 mg/kg) group (Table 1).

#### Effect on RSV on spontaneous motility

Spontaneous motility was progressively deteriorated after 7th day of COL administration, with maximum deterioration on 21st day [F_(6,28)_ = 9.973 (*p* < 0.05)]. RSV administration failed to show any effect on 7th and 14th day. However, oral administration of RSV (5, 10 & 20 mg/kg) showed significant improvement in scoring on 21st day (Table 1).

#### Effect of RSV on forelimb flexion

Forelimb flexion was significantly deteriorated in COL treated rats as compared to control group [F_(6,28)_ = 5.788 (*p* < 0.05)]. RSV (5 and 10 mg/kg) did not show any improvement in forelimb flexion as compared to COL treated groups. However, RSV (20 mg/kg) showed significant restoration of forelimb flexion from 14th day onwards as compared to ICV-COL treated group as well as RSV (5 and 10 mg/kg) treated group (Table 1).

#### Effect of RSV on righting reflex

Righting reflex in animals was progressively lost from 7th day with maximum reduction on 14th day (Table 1). There was statistically significant decrease in righting reflex in COL treated rats as compared to the control rats [F_(6,28)_ = 5.142 (*p* < 0.00)]. Oral administration of RSV (20 mg/kg) showed significant improvement in righting reflex as compared to COL treated rats. However, RSV (5 & 10 mg/kg) did not show any improvement in the righting reflex (Table 1).

### Effect of RSV (5, 10 and 20 mg/kg) on behavioral paradigms in ICV-COL injected rats

#### Effect of RSV on ICV-COL induced immobility

**Fig. 2:**
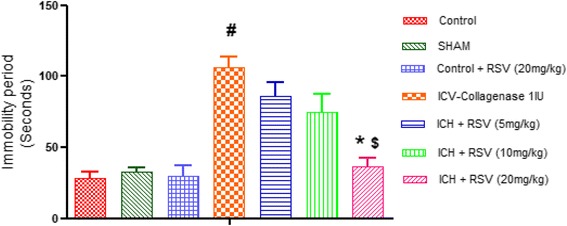
Effect of RSV (5, 10 and 20 mg/kg) on immobility time in forced swim test in ICV-COL treated rats. Values are expressed as mean ± SEM. ICH indicates ICV-COL treated rats. One way ANOVA followed by Tukey’s multiple comparison tests was applied. #*p* < 0.05 as compared to control group. *p < 0.05 as compared to ICV-COL group. $*p* < 0.05 as compared to ICH + RSV (5 mg/kg) group

Porsolt forced swim test was used to evaluate post stroke depression in animals. Depressive behavior is positively correlated with the immobility time. COL administration lead to significant increase in immobility time (139.0 ± 22.05 s) as compared to the control group (16.6 ± 6.2 s) [F_(6,28)_ = 15.89 (*p* < 0.05)]. Compared to ICV-COL group, RSV (20 mg/kg) showed significant reduction in immobility time (Fig. 2). However, there was no effect with RSV (5 and 10 mg/kg). Furthermore, RSV (20 mg/kg) produced significantly more pronounced effect as compared to RSV (5 mg/kg).

#### Effect of RSV on COL-induced in-coordination in muscular strength

**Fig. 3:**
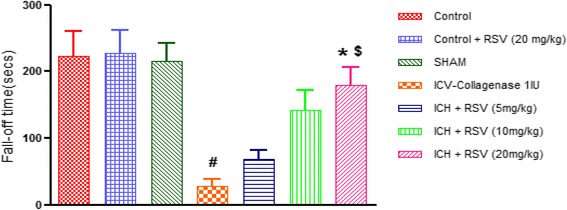
Effect of RSV (5, 10 and 20 mg/kg) on muscular strength in Rota rod test. Values are expressed as mean ± SEM. ICH indicates ICV-COL treated rats. One way ANOVA followed by Tukey’s multiple comparison tests was applied. ^#^ *p* < 0.05 as compared to control group. *p < 0.05 as compared to ICV-COL group. ^$^ *p* < 0.05 as compared to ICH + RSV (5 mg/kg) group

Rota rod test was done to evaluate post-stroke impairment in muscular activity on 21st day. As per a previous study done in our lab, we found maximum reduction in locomotor activity at 21st day after induction of stroke. At 21st day, fall-off time was significantly lowered in ICH group as compared to control [(F_(6,28)_ = 8.33 (*p* < 0.05)]. Treatment with RSV (20 mg/kg) significantly increased fall-off time as compared to COL treated rats (Fig. 3). Per se administration of RSV (20 mg/kg) did not show any difference from control group.

#### Effect of RSV on COL-induced locomotor dysfunction

**Fig. 4:**
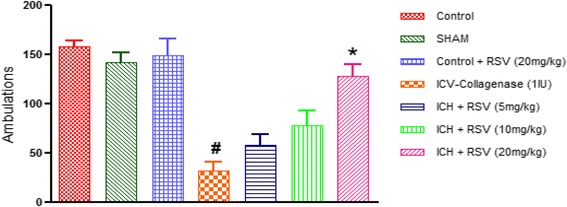
Effect of RSV (5, 10 and 20 mg/kg) on locomotor activity on 21th day after ICV-COL administration in rats. Values are expressed as mean ± SEM. ICH indicates ICV-COL treated rats. One way ANOVA followed by Tukey’s multiple comparison tests was applied. #*p* < 0.05 as compared to naïve group. *p < 0.05 as compared to ICV-COL group

Locomotor dysfunction related to post stroke neurological deficits was tested by a digital actophotometer. As per previous study done in our lab, we found maximum change in locomotor activity on 21st day after stroke induction. On day 21st, locomotor activity was significantly decreased in ICV-COL group than control group [(F_(6,28)_ = 17.66) (*p* < 0.05)]. RSV (20 mg/kg) showed significant increase in ambulatory activity than ICV-COL group (Fig. 4), although RSV (5 and 10 mg/kg) did not show any effect. Sham group did not produce any effect as compared to the control group. Furthermore, per se treatment of RSV (20 mg/kg) did not show any difference from control.

#### Effect of RSV on mechanical hyperalgesia and tactile allodynia

**Fig. 5:**
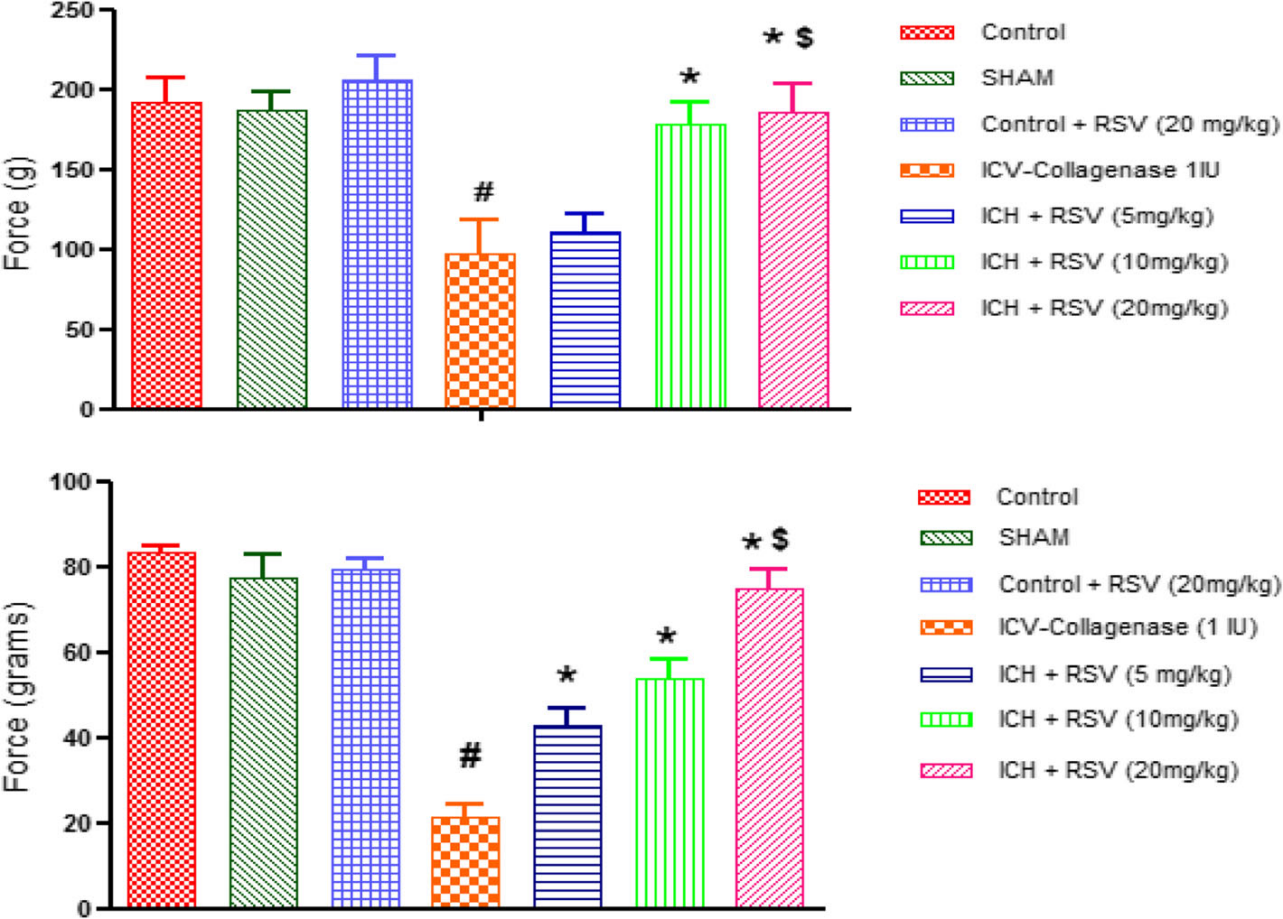
**a** Effect of RSV (5, 10 and 20 mg/kg) on mechanical hyperalgesia in ICV-COL treated rats. **b** Effect of RSV (5, 10 and 20 mg/kg) on tactile allodynia in ICV-COL treated rats. Values are expressed as mean ± SEM. ICH indicates ICV-COL treated rats. One way ANOVA followed by Tukey’s multiple comparison tests was applied. *p < 0.05 as compared to ICV-COL. #*p* < 0.05 as compared to control group. $*p* < 0.05 as compared to ICH + RSV (5 mg/kg)

Paw withdrawal threshold in the Randall-Sellito test was reduced by 54% in COL treated rats as compared to control rats [F_(6,28)_ = 8.52 (*p* < 0.05)] (Fig. 10). Tactile withdrawal threshold in response to light touch with flexible von-Frey filaments was also reduced by 74% in COL treated group as compared to control group [F_(6,28)_ = 21.13 (*p* < 0.05)] (Fig. 10) on 21st day after COL administration. Oral administration of RSV (10 and 20 mg/kg) significantly restored paw withdrawal threshold in Randall-Sellito test, but RSV (5 mg/kg) did not show any effect (Fig. 5a). RSV (20 mg/kg) showed statistically significant difference from RSV (5 mg/kg). In von-Frey filament test, RSV (5, 10 & 20 mg/kg) showed statistically significant increase in paw withdrawal threshold (Fig. 5b).

### Effect of RSV on different parameters in MWM test

#### Effect of RSV on escape latency time (sec)

The cognitive function and memory consolidation was estimated using MWM test from 23rd to 27^th^ day. There was no difference in the mean escape latency in any of the group on first and second day. However, from 25th day onwards, there was a progressive decline in mean escape latency time. COL treated rats showed significant reduction [F_(6,28)_ = 10.48 (*p* < 0.05)] in escape latency as compared to the control group (Table 2). This poor performance in memory was significantly mitigated [F_(6,28)_ = 10.48 (*p* < 0.05)] by treatment with RSV (20 mg/kg) on 25th, 26th and 27th day of the training and with RSV (10 mg/kg) on 27th day. However, RSV (5 mg/kg) did not show any effect.

**Table 2:** Effect of RSV (5, 10 and 20 mg/kg) on escape latency (sec)

Groups	Day 23	Day 24	Day 25	Day 26	Day 27
Control	85.6 ± 2.55	72.4 ± 7.99	41.2 ± 9.20	36.4 ± 4.74	13.2 ± 3.59
SHAM	83.2 ± 4.90	74.2 ± 5.87	44.2 ± 9.46	44.6 ± 11.29	21.8 ± 4.21
Control + RSV (40 mg/kg)	83.9 ± 2.97	65.4 ± 6.07	42.6 ± 9.23	25.4 ± 4.64	11.6 ± 4.4
ICV-Collagenase (1 IU)	87.4 ± 1.78	80.8 ± 4.37	75.2 ± 5.32^#^	69.4 ± 9.42^#^	65.6 ± 10.32^#^
ICH + RSV (5 mg/kg)	83.6 ± 4.23	80.4 ± 5.07	60.8 ± 8.08	48.2 ± 11.55	40.7 ± 12.4
ICH + RSV (10 mg/kg)	79.6 ± 8.77	72.4 ± 9.04	49.3 ± 11.63	35.8 ± 9.23	26.8 ± 5.26*
ICH + RSV (20 mg/kg)	81.6 ± 8.17	66 ± 6.56	32.6 ± 6.45*	22.8 ± 2.74*	16.4 ± 2.78*

Values are expressed as mean ± SEM. ICH indicates ICV-COL treated rats; RSV, resveratrol. One way ANOVA followed by Tukey’s multiple comparison tests was applied. #*p* < 0.05 as compared to control group. *p < 0.05 as compared to ICV-COL group. ^$^ *p* < 0.05 as compared to ICH + RSV (5 mg/kg)

#### Effect of RSV on total distance travelled to reach the hidden platform (path length in cm)

**Fig. 6:**
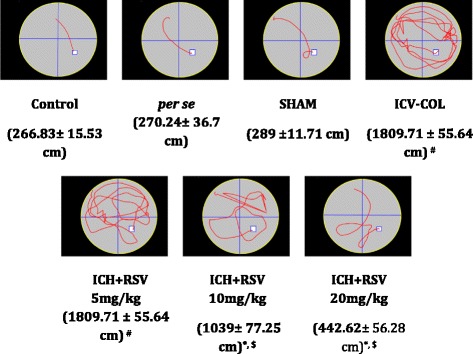
Effect of RSV (5, 10 and 20 mg/kg) on total distance travelled in Morris water maze test (path length in mean ± SEM cm). ICH indicates ICV-COL treated rats; RSV, resveratrol. One way ANOVA followed by Tukey’s multiple comparison tests was applied. ^#^ *p* < 0.05 as compared to control group. *p < 0.05 as compared to ICV-COL group. ^$^ *p* < 0.05 as compared to ICV + RSV (5 mg/kg) group

Progressive increase in path length to reach the hidden platform on subsequent days in MWM test is associated with memory disruption. There was no difference among various groupsin total distance travelled on 23^rd^and 24^th^day. From 25th day onwards, there was a significant difference in path length in COL treated group as compared to the control group [F_(6,28)_ = 10.48 (*p* < 0.05)] (Fig. 6). Treatment with RSV (10 and 20 mg/kg) significantly decreased path length as compared to the COL treated group, suggesting improvement in memory. However, no significant effect was shown by RSV (5 mg/kg).

#### Effect of RSVon time spent in target quadrant in MWM test

**Fig. 7:**
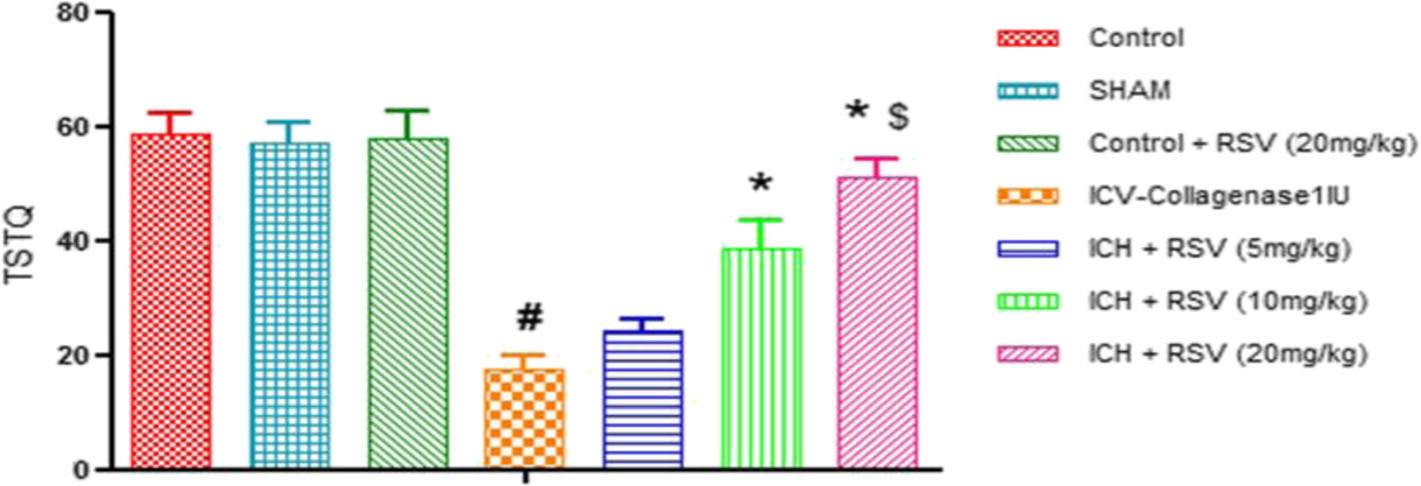
Effect of RSV (5, 10, and 20 mg/kg) on time spent in target quadrant. Values are expressed as mean ± SEM. ICH indicates ICV-COL treated rats; TSTQ, time spent in target quadrant. One way ANOVA followed by Tukey’s multiple comparison tests was applied. #*p* < 0.05 as compared to naïve group. *p < 0.05 as compared to ICV-COL group. $*p* < 0.05 as compared to ICH + RSV (5 mg/kg) group

To assess how well the animals had learned and consolidated the plateform location during the training, probe trial was carried out by removing the platform and time spent by the animal in target quadrant (one previously containing platform) was recorded. The time spent in target quadrant was significantly lowered in COL treated rats as compared to control rat [F_(6,28)_ = 22.70 (*p* < 0.05)]. Oral treatment with RSV (10 and 20 mg/kg) significantly increased time spent in the target quadrant as compared to COLtreated rats (Fig. 7). However, RSV (5 mg/kg) did not show any effect.

### Effect of RSV on different biochemical parameters

#### Effect of RSV on COL-induced changes in lipid peroxidation levels

**Fig. 8:**
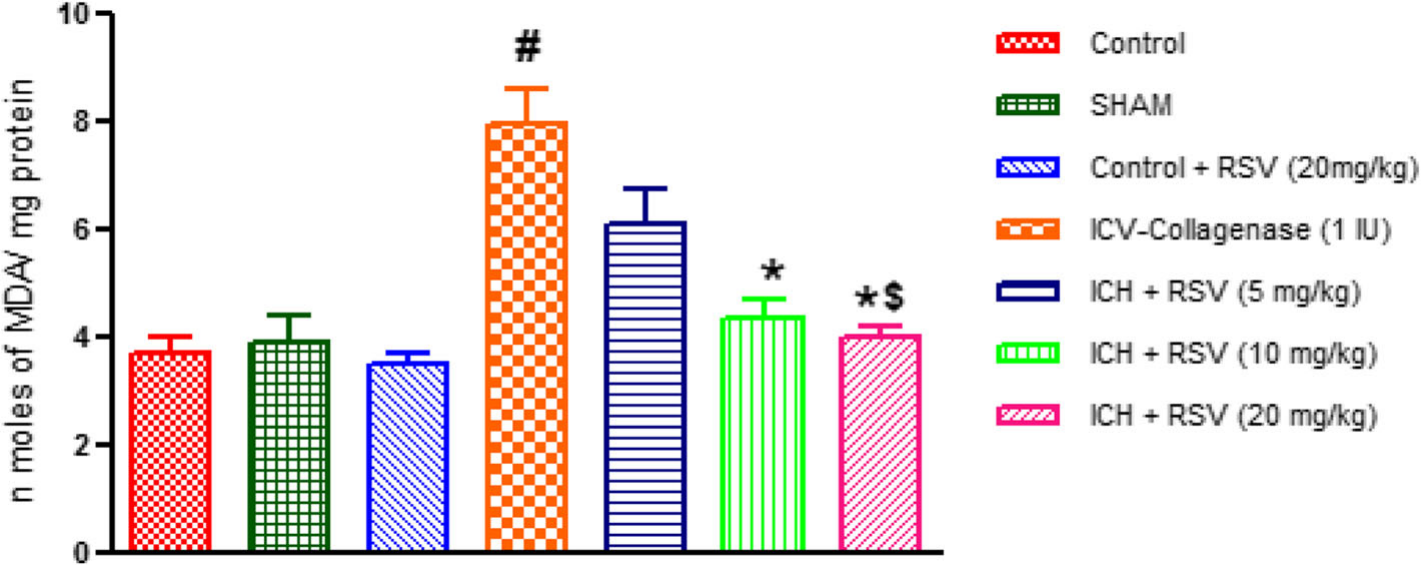
Effect of RSV (5, 10 and 20 mg/kg) on lipid peroxidation levels in ICV-COL treated rats. Values are expressed as mean ± SEM. ICH indicates ICV-COL treated rats; RSV, RSV. One way ANOVA followed by Tukey’s multiple comparison tests was applied. #*p* < 0.05 as compared to control group. *p < 0.05 as compared to ICV-COL group. $ < 0.05 as compared to ICH + RSV (5 mg/kg) group

Thiobarbituric acid reactive substances (TBARS) levels were significantly increased in the brain of COL treated rats as compared to control [F_(6,28)_ = 13.97 (*p* < 0.05)]. Treatment with RSV (10 & 20 mg/kg) significantly decreased TBARS levels as compared to COL treated rats. However, sham and per se groups did not show any difference as compared to control group (Fig. 8).

#### Effect of RSV on catalase, glutathione and superoxide dismutase levels

**Fig. 9:**
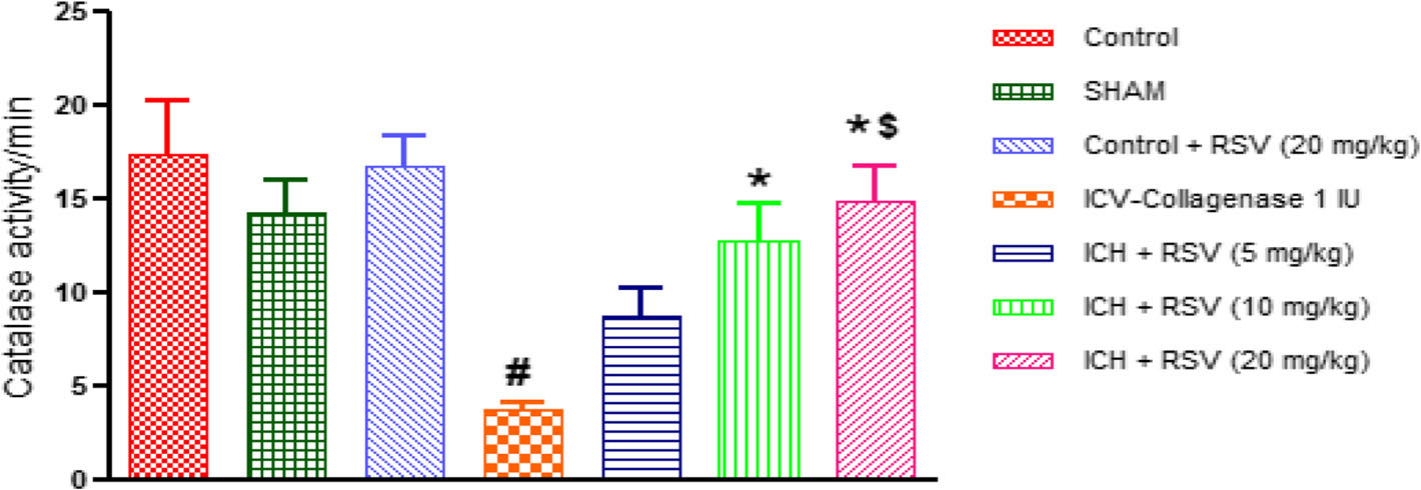
Effect of RSV (5, 10 and 20 mg/kg) on catalase activity in ICV-COL treated rats. Values are expressed as mean ± SEM. ICH indicates ICV-COL treated rats; RSV, RSV. One way ANOVA followed by Tukey’s multiple comparison tests was applied. #*p* < 0.05 as compared to control group. *p < 0.05 as compared to ICV-COL group. $ < 0.05 as compared to ICH + RSV (5 mg/kg) group

**Fig. 10:**
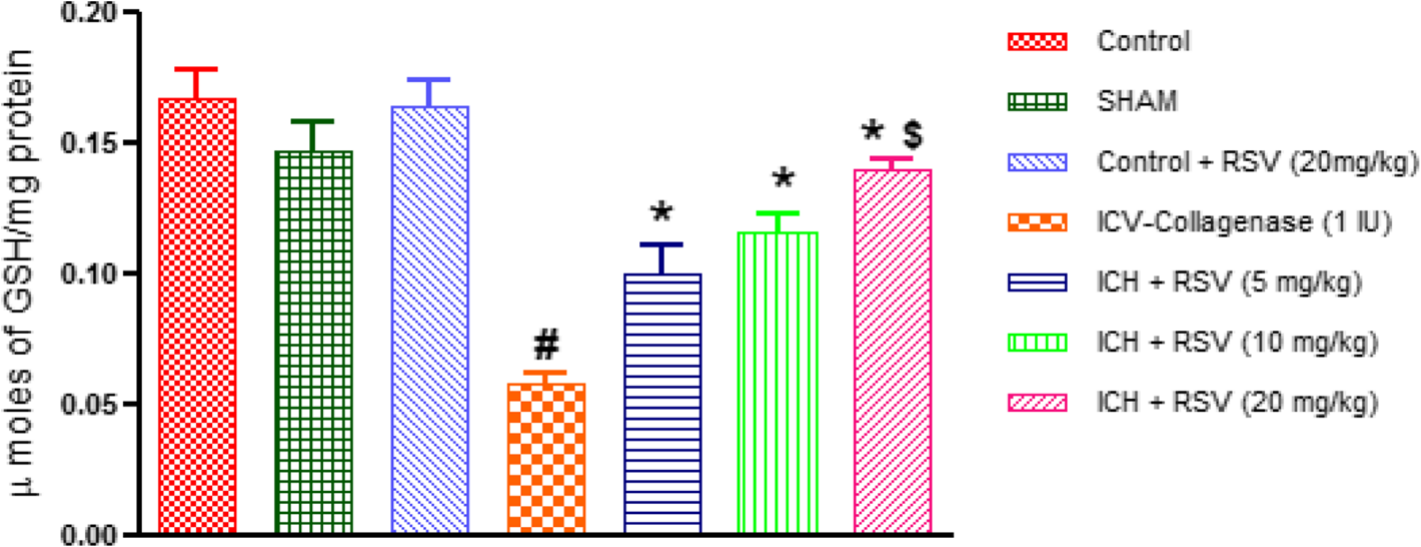
Effect of RSV (5, 10 and 20 mg/kg) on reduced glutathione (GSH) levels in ICV-COL treated rats. Values are expressed as mean ± SEM. ICH indicates ICV-COL treated rats; RSV, RSV. One way ANOVA followed by Tukey’s multiple comparison tests was applied. ^#^ *p* < 0.05 as compared to control group. *p < 0.05 as compared to ICV-COL group. ^$^ *p* < 0.05 as compared to ICH + RSV (5 mg/kg) group

**Fig. 11:**
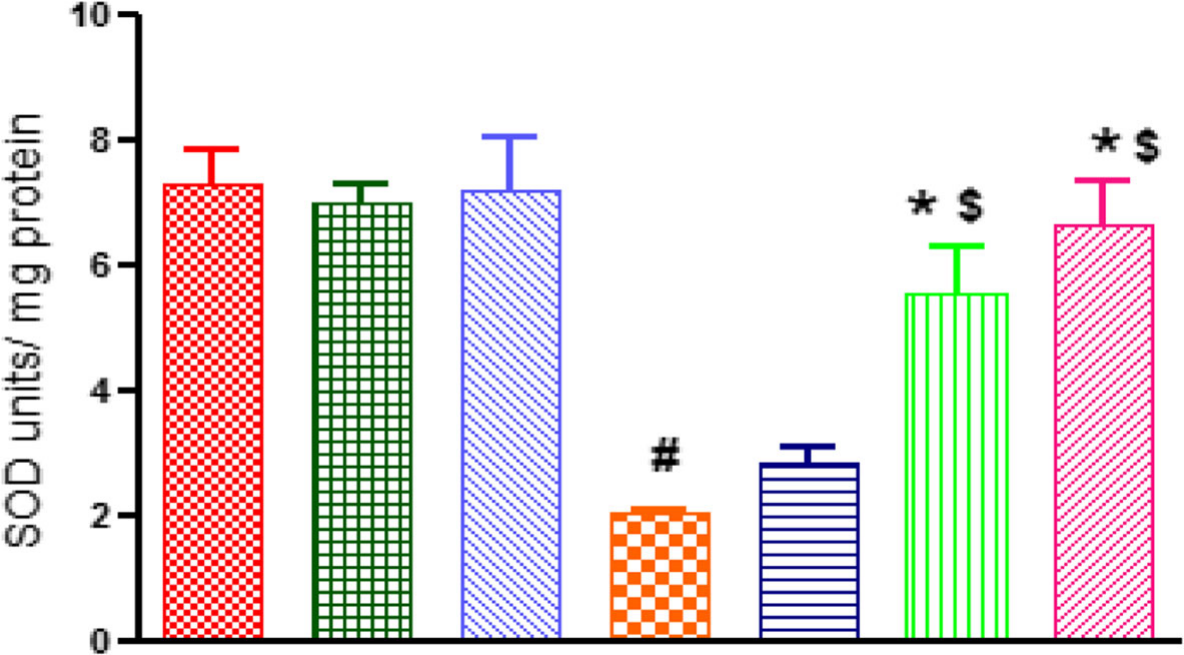
Effect of RSV (5, 10 and 20 mg/kg) on superoxide dismutase activity in ICV-COL treated rats. Values are expressed as mean ± SEM. ICH indicates ICV-COL treated rats; RSV, RSV. One way ANOVA followed by Tukey’s multiple comparison tests was applied. . #*p* < 0.05 as compared to control group. *p < 0.05 as compared to ICV-COL group. $ *p* < 0.05 as compared to ICH + RSV (5 mg/kg) group

The reduced glutathione levels and enzymatic activity of catalase and superoxide dismutase were significantly decreased in the brains of COL treated rats as compared to control rats. This reduction was significantly (*p* < 0.05) restored by oral administration of RSV (10 and 20 mg/kg). However, RSV (5 mg/kg) showed improvement in reduced glutathione level only (Figs. 9, 10 and 11).

#### Effect of RSV on nitrite levels

**Fig. 12:**
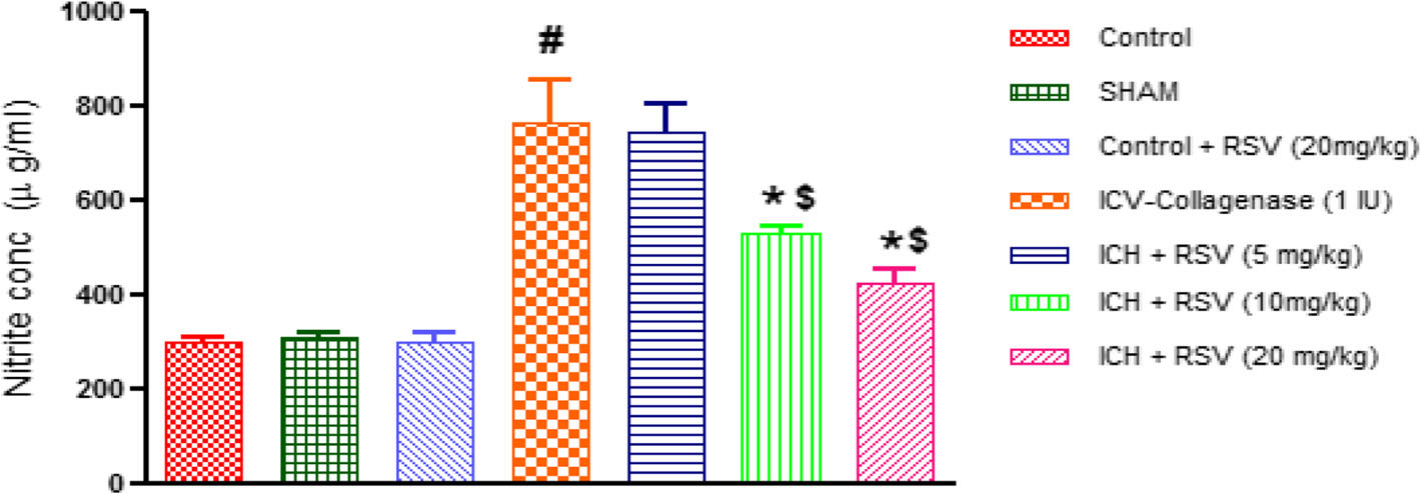
Effect of RSV on nitrite levels in ICV-COL treated rats. The values are expressed as mean ± SEM. ICH indicates ICV-COL treated rats; RSV, RSV. One way ANOVA followed by Tukey’s multiple comparison tests was applied. ^#^ *p* < 0.05 as compared to control group. *p < 0.05 as compared to ICV-COL group. ^$^ *p* < 0.05 as compared to ICH + RSV (5 mg/kg) group

Nitrite levels were significantly increased in ICV-COL treated rats as compared to control [F_(6,28)_ = 22.12 (*p* < 0.05)]. Oral administration of RSV (10 & 20 mg/kg) significantly decreased nitrite levels as compared to the COL treated rats, but RSV (5 mg/kg) did not show any effect (Fig. 12). RSV (10 and 20 mg/kg) showed significant decreased in nitrite level when compared to RSV (5 mg/kg) group. However, sham and per se groups did not show any difference as compared to control animals.

#### Effect of RSV on TNF-α levels

**Fig. 13:**
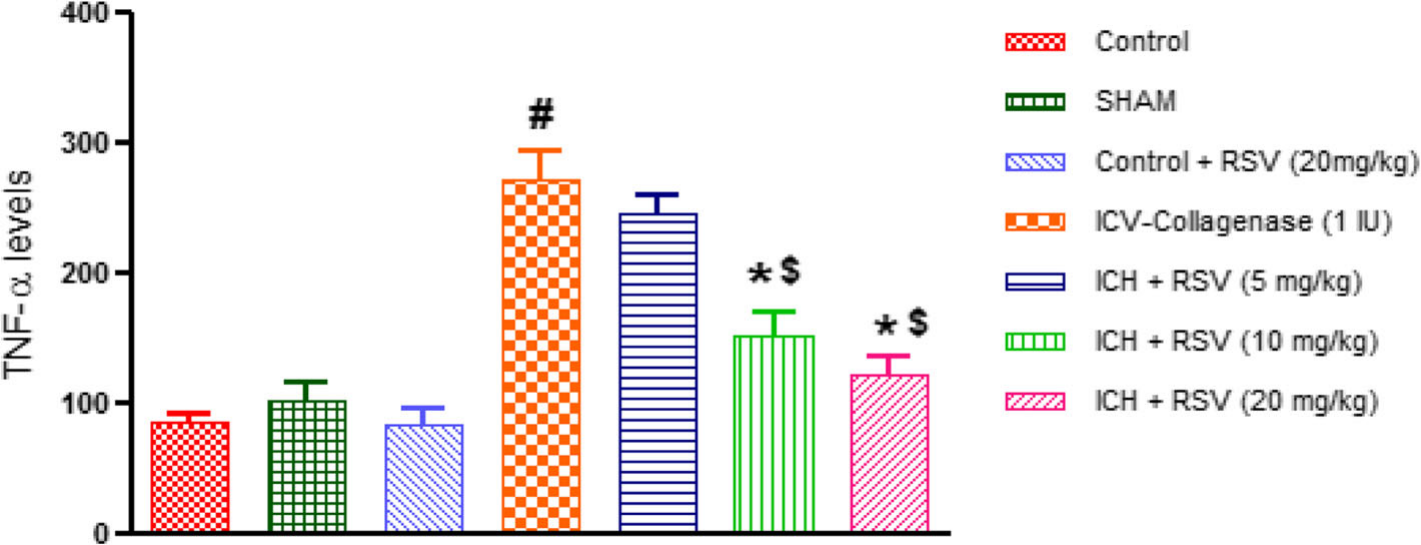
Effect of RSV (5, 10 and 20 mg/kg) on pro-inflammatory cytokine TNF-alpha level in ICV-COL treated rats. Values are expressed as mean ± SEM. ICH indicates ICV-COL treated rats; RSV, RSV. One way ANOVA followed by Tukey’s multiple comparison tests was applied. ^#^ *p* < 0.05 as compared to control group. *p < 0.05 as compared to ICV-COL group. ^$^ *p* < 0.05 as compared to ICH + RSV (5 mg/kg) group

Single ICV-COL administration (0.25 IU) significantly increased pro-inflammatory cytokine, TNF-alpha, in the rat brain as compared to control [F_(6,28)_ = 25.50 (*p* < 0.05)]. RSV treatment (10 and 20 mg/kg) significantly attenuated TNF-alpha level as compared to ICV-COL group, but RSV (5 mg/kg) did not show any effect (Fig. 13). However, RSV (10 and 20 mg/kg) showed significant decreased as compared to RSV (5 mg/kg).

## Discussion

ICV-COL model is a reliable and reproducible ICH model. It has been previously studied that COL injection in brain increases blood brain barrier permeability and brain edema which further leads to cognitive and behavioral deficits in animals [44, 45]. Furthermore, basal lamina destruction and erythrocytic lysis induced by COL leads to increased generation of free radicals, pro-inflammatory cytokines, neuroinflammation, neuronal cell death and oxidative stress as seen in secondary injury during ICH.

Movement and functional disorders are seen in about 0.08% of the stroke patient causing temporary to permanent disability [46]. The mechanism leading to stroke related functional disorders is not clear, but probably it is the delayed phase hematoma expansion induced damage to striatal neurons [47] and resulting dopamine loss in the basal ganglia. Strial neurons act as relay centre for neuronal connections in basal ganglia. This hypothesis can be further corroborated by the fact that levodopa treatment improves functional recovery after experimental stroke [48]. Moreover, brain herniation and oxido-inflammation caused by delayed ischemia in hemorrhagic stroke can cause focal damage and loss of function in particular brain region. Hypokinetic movement disorders are well reported in unilateral or bilateral infarction in striatum and lentiform nucleus [49, 50]. In our study, we performed neurological scoring by using a battery of tests like horizontal bar test, forelimb flexion, righting reflex, cylinder test, spontaneous motility [31]. Actophotometer test and rotarod test were done to assess motor impairments. We found that ICV COL administration produced significant motor and neurological defects, probably caused by acute caudate vascular lesion and oxido-inflammatory cerebral injury. Antioxidants have been shown to attenuate neuromotor deficits associated with various neurodegenerative diseases in different animal models [51–53]. RSV restored these motor and neurological deficits owing to its inhibitory effect on oxido-inflammatory cascade involved in secondary brain injury after ICH.

Free radical generation and oxidative stress are the two most important factors in the ICH related brain injury. In the present study, an increase in oxidative stress is indicated by increased malondialdehyde and nitrite levels along with decreased endogenous antioxidants like catalase, superoxide dismutase and reduced glutathione levels. Vital organs like brain are more vulnerable to ischemic damage and oxidative stress due to its paucity of antioxidant defenses, uninterrupted oxygen demand and high lipoic myelin and iron content. Moreover, several compounds with antioxidant properties have been demonstrated to reduce stroke-related brain damage in animal models. Furthermore, delayed cerebral ischemia due to hematoma obstruction activates calcium dependent nitric oxide synthase which leads to increase in nitric oxide and ROS generation, leading to blood brain barrier disruption. This oxido-nitrosative stress leads to major neurological deficits like dementia, pain and depression in COL treated rats. In the present study, we found that RSV attenuated oxidative stress in the brain of COL treated rat by virtue of its strong antioxidant potential. RSV has been found to show antioxidant property by virtue of its free radical scavenging activity to protect hippocampal neuronal cells against toxicity induced by nitric oxide [54]. Moreover, RSV up regulates heme-oxygenase 1 (HO1), an endogenous anti-oxidant protecting against neuronal cell death [55].

Microglia is activated within minutes after ICH to clear hematoma and cell debris by phagocytosis. However, emerging evidences indicate that activated microglia contribute to hemorrhage related cell damage by releasing different inflammatory cytokines including TNF-α. TNF-α has been shown to increase after ICH in different in-vivo and in-vitro studies [56–58]. The evidence is further strengthened by the fact that inhibition of microglia decreases ICH related brain damage [59]. Plasma TNF-α level has been shown to have direct correlation with brain edema in ICH patients [60]. In the present study, inflammatory cytokine generation as evident by elevated levels of TNF-α, was inhibited by RSV (10 & 20 mg/kg). Our observation is supported by the study done by Bi XL in which RSV inhibits nitric oxide and TNF-α production by lipopolysaccharide-activated microglia has also been reported to inhibit TNF-α both in-vivo and in-vitro [61].

Physical disability, cognitive impairment and social isolation are the common factors leading to post stroke depression, anhedonia, anxiety and mood disorders and are seen in about one third of the stroke patients [62–64]. In the present study, post stroke depression was evaluated using forced swim test. COL-induced post stroke depression is evident by increase in immobility time in force swim test on 14th day after COL administration in ICV-COL group. Increase oxidative stress and inflammatory cytokines in COL-induced ICH leads of depressive symptom. Antioxidant and anti-inflammatory agents from natural resources have been tried successfully in the amelioration of depressive behavior in different animal models [65–68]. Moreover, extensive data on anti-depressant action of RSV has been reported [69–71]. RSV has also been reported to inhibit monoamine oxidase activity (MAO) in vitro [72] and to up regulate serotonin, dopamine and nor-epinephrine in a mice model of depression [73]. Moreover, RSV has been shown to regulate HPA-axis and provide beneficial effect in ischemic stroke associated depression [70]. In our study RSV at highest dose (20 mg/kg) restores the increased in immobility time in FST which is supported by the study done by Xu et al., [73] where *trans-*RSV led to a dose-dependent reduction in the immobility period.

As ICH leads to progressive memory deterioration and cognitive decline, MWM test was used to assess memory function. Increased escape latency (time spent to locate hidden platform) in repeated trials demonstrated memory deficit. Mean distance travelled was significantly reduced in RSV treated group as compared to the COL treated rats. Our observation is supported by the study in which RSV improves cognition and reduces oxidative stress in rats with vascular dementia [74]. Previous studies have potentiated the role of neuroinflammation and microglial activation in memory deterioration and cognitive decline. RSV improved memory and cognition in ICV colchicine-induced cognitive impairment [75]. RSV also inhibits LPS challenged microglial nitric oxide production by inhibiting NF-κB as evident by an in-vitro study [61, 76]. Moreover, RSV has shown potent anti-inflammatory and neuroprotective effects in different models of ischemic stroke [23, 77, 78].

Central post stroke pain (CPSP) is a pain syndrome common among 2–8% of stroke patients [79]. Neuropathic pain is mainly associated with brain lesion or disease of somatosensory system [80]. The mechanism of CPSP is still ambiguous but thought to be involvement of neuroinflammation induced activation of cell surface purinergic receptors [81]. P_2_X_7_ receptor, a type of purinergic receptors is widely involved in pro-inflammatory receptors effects in central nervous system [82]. Subsequent release of inflammatory cytokines like IL-1β has been proved to sensitize and destroy nerve terminals, resulting in neuropathic pain [83]. ICV COL reduced pain threshold in von Frey hair and Randall-Selitto by releasing inflammatory cytokines like TNF-α and IL-1β. There was a significant increase in paw withdrawal threshold in RSV administered animals as compared to the COL treated animals [84, 85].

## Conclusion

In the present study, we found that RSV exerts anti-oxidant and neuroprotective effects against COL-induced oxidative-stress and neuronal deficits, possibly by strengthening endogenous anti-oxidant machinery. A significant decrease in TNF-α levels and nitrosative stress corroborate our findings related to protective effects against inflammatory cytokine and nitrosative stress induced neuronal cell death and post stroke complications.

## Abbreviations

COL: COL
CPSP: Central post stroke pain
FST: Forced swim test
GSH: Reduced gluthathione
i.p.: Intraperitoneal
IAEC: Institutional animal ethics committee
ICH: Intracerebral hemorrhage
ICV: Intracerebroventricular
IU: International unit
LPO: Lipid peroxidation
MAO: Monoamine oxidase
MMP-9: Matrix metalloproteases-9
MWM: Morris water maze
NBT: Nitro blue tetrazolium
p.o.: Per oral
ROS: Reactive oxygen species
RSV: Resveratrol
SOD: Superoxide dismutase
TBARS: Thiobarbituric acid reactive substances
TNF-α: Tumor necrosis factor α
